# Silent Suffering: The Tragic Suicide Among Medical Residents

**DOI:** 10.34172/aim.2024.41

**Published:** 2024-05-01

**Authors:** Ali Montazeri, Sedigheh Hantoushzadeh, Seyed Jafar Razavi, Mohadese Dashtkoohi, Nasim Eshraghi, Marjan Ghaemi

**Affiliations:** ^1^Health Metric Research Center, Iranian Institute for Health Sciences Research, ACECR, Tehran, Iran; ^2^Vali-E-Asr Reproductive Health Research Center, Family Health Research Institute, Tehran University of Medical Sciences, Tehran, Iran

## Dear Editor,

 We are writing to draw attention to the high suicide rate among medical residents – a phenomenon exacerbated by the challenging conditions faced by residents in Iran. Suicide, as a major public and professional health issue, is remarkably higher among physicians-in-training, with an estimated incidence rate of 11‒20 per 100 000 person-years, indicating that medical residency itself could be considered a risk factor for suicide.^1^ Suicide ranks as the second most prevalent cause of death among residents. Specifically, it stands as the primary cause of mortality among male residents and the second most common cause among female residents.^2^

 There is substantial diversity in suicide rates depending on the field and the country of education. In this regard, in a survey conducted in the United States, psychiatry residents had the highest suicide rates. In contrast, the lowest rates were found among dermatology residents.^3^

 As reported by Yaghmour et al, a higher percentage of suicide deaths occurred during the first and third quarters of the academic year as well as during the early phases of residency training. However, there was no evidence of a particular trend in the number of suicides among residents throughout the study. It is equally concerning that the suicide rate among medical residents has not shown a decline over time, leading to the conclusion that our efforts are falling short of what is required.^4^

 This trend is different for graduated physicians. Since 1980, female physicians have had a higher standardized mortality ratio (SMR) for suicide than male physicians. Nevertheless, it has been observed that the suicide SMR has decreased in both groups over time.^5^ It indicates that the situation becomes more serious during training than after graduation.

 Burnout and depression are prevalent and widely recognized issues among medical residents, and numerous studies have demonstrated a clear correlation between these conditions and increased risk of suicide.^1^ Approximately 20% of medical residents meet the criteria for depression, whereas the percentage rises to 74 when it comes to burnout.^1^ In addition to the pressure of completing medical school and incurring debt, the demanding nature of the job can also contribute to burnout.^6^

 The situation of suicide among Iranian medical residents is particularly dire. According to reports from the Medical Council of Iran,^7^ within the past 12 months alone, 16 residents have tragically taken their own lives. Medical residency in Iran differs extensively from other countries, primarily due to disparities in salary and post-graduation work opportunities. Currently, the salary of a medical resident in Iran averages around $2400 annually, in contrast to the mean salary of $55 000 for US medical residents or $40 000 for UK medical residents or around $58 000 per year for medical residency in Germany. Furthermore, postgraduate work often involves placements in remote and difficult-to-reach hospitals. Additionally, first-year residents in Iran frequently endure exhausting schedules, with shifts every other day equating to 36 hours of continuous work.^8^

 Even though suicide is only the tip of the iceberg in terms of mental illnesses and the environmental factors that lead to this tragic event, the true extent of the problem remains unclear.

 In addition, as a result of the SARS-CoV-2 pandemic and the subsequent COVID-19 crisis, mental health problems are expected to rise abruptly among all healthcare workers. This dramatic escalation can be due to several factors, including expanded work hours, social isolation, fewer opportunities for self-care, and higher exposure to emotionally traumatic events in both workplace and home.^9^

 The matter under discussion which pertains to why a healer turns into someone who takes their own life is a complex issue with no simple solution to it. We must, however, join forces to address this critical matter and try to support those in need. Interventions such as stress reduction through mindfulness and fostering a culture of support may help decrease the rate of suicide among medical residents.^10^ This can be achieved through mentorship programs, peer support groups, and easier access to mental health resources. Notably, the residents who had a mentor were less likely to report burnout and suicidal ideation.^11^ Furthermore, it is crucial to promote the adoption of policies and practices within healthcare institutions that facilitate changes in the residency system. These changes should prioritize the well-being of medical residents by reducing work hours, addressing issues of discrimination and harassment in the workplace, and ensuring the provision of support. Early recognition of the individuals who may become suicidal, prior to the start of the residency period and providing more support to them is also a matter of great importance.^12^

 Future research should focus on developing targeted system-based approaches tailored to medical residents’ needs to combat suicide effectively.

 In conclusion, the high incidence of suicide among medical residents is a major public health concern that necessitates continued investigation and effective interventions. We urge all healthcare institutions, policymakers, and researchers to work together to prevent this dreadful event.
